# An Overview of Female Genital Mutilation in Africa: Are the Women Beneficiaries or Victims?

**DOI:** 10.7759/cureus.10250

**Published:** 2020-09-04

**Authors:** Ganiyu O Shakirat, Muhammad A Alshibshoubi, Eldia Delia, Anam Hamayon, Ian H Rutkofsky

**Affiliations:** 1 Obstetrics and Gynecology, California Institute of Behavioral Neurosciences & Psychology, Fairfield, USA; 2 Internal Medicine, California Institute of Behavioral Neurosciences & Psychology, Fairfield, USA; 3 Psychiatry, Neuroscience, Internal Medicine, California Institute of Behavioral Neurosciences & Psychology, Fairfield, USA

**Keywords:** fgm, who, dhs

## Abstract

Female Genital Mutilation (FGM) is a social phenomenon that is deeply rooted in African socio-cultural and religious facets. It covers a sequence of procedures carried out on the genitals of females of different ages, including total or partial removal of the female external genitalia or other injuries to the female genital organs for non-medical reasons. Several studies have shown beyond a reasonable doubt that FGM is more of a detriment than benefit to the mutilated women. Hence, this review comprehensively presents the narratives and experience of African women about FGM with a focus on whether they are beneficiaries or victims of the practice.

The method adopted involved searching for relevant studies through PubMed and Google Scholar databases coupled with some prominent internet materials. This method was done majorly to identify and utilize the best quality published studies on FGM in Africa.

Having lent due credence to the relevant studies pooled together, it was established that the practice of FGM in the African continent is highly undesirable. It creates numerous health complications coupled with psychosocial, psychological, and psychosexual issues for the mutilated women. Prominent among these repercussions are infection, the formation of scarring and keloid, monthly menstrual difficulties, urinary symptoms, infertility, obstetric complications during pregnancy and labor, depression, anxiety, and post-traumatic stress disorder. Despite these complications, it was revealed that FGM is still extant in many African countries. However, the fear of becoming a social outcast is the biggest hindrance facing anti-FGM campaigners.

FGM is unequivocally a cankerworm that has eaten the freedom and wellbeing of African women, making them choiceless victims. Therefore, it is quite pertinent for governments and community leaders to provide medical support to the victims and fight the prevalence of FGM in their jurisdictions. This can be achieved through increased awareness about its adverse effects, women's education, and community shunning of the practice coupled with appropriate sanctioning of the erring members.

## Introduction and background

Female genital mutilation (FGM), otherwise regarded as female circumcision (FC), has long been a common phenomenon, mentioned even by Greek geographers and historians such as Strabo (64 BC-23 AD) and Herodotus (425-484 BC). It refers to any procedure involving complete or partial removal of external female genitalia or any other injury to their genitals for reasons aside from medical indications [1]. The occurrence of FGM among the Egyptians along the Nile Valley during the reign of the Pharaohs has led to the proposition that Egypt is the source country of the practice [2]. Similarly, FGM has also been testified to some centuries ago among other nations, especially in Rome, where it was being practiced majorly to safeguard their slaves from unwanted pregnancies [3]. Although religious scholars have asserted that the practice has no backing in the holy books, FGM spans ethnicities and religious backgrounds. It has been banned in the Western world; however, immigrant populations from Africa, Asia, the Pacific, and parts of the Middle East have a high proportion of circumcised females [4]. In a 2016 report, Teixeira and Lisboa found that in Portugal, there might be more than 6,500 immigrant females 15 years or older who have been circumcised and 1,830 girls under 15 years who are probably going to or have experienced circumcision [4].

In the medical context, FGM comprises four major types. Type I, otherwise known as clitoridectomy, involves total or partial removal of the clitoris; Type II, or excision, entails cutting either partially or wholly of the clitoris as well as the labia minora and/or labia majora; Type III, or infibulation, involves the narrowing of the vaginal orifice and creating a covering seal; whilst Type IV connotes any other harmful practice to the female genitalia, for instance, piercing, scraping or pricking [5]. Regardless of the type, even though it is part of indigenous cultures across prominent African tribes, it has been equally referred to as a well-known violation of women's rights and an extreme form of discrimination against women. In essence, it undermines the rights of these women with respect to their health and security as well as physical integrity. More so, it exposes them to forms of torture and inhuman treatment which might also result in death [6].

FGM has been traced to a range of medical, sociocultural, economic, and women rights consequences. These have triggered global and multifaceted attention towards its eradication, albeit with slow progress over the years [7]. Female genital mutilation is currently practiced in almost 30 countries in Africa and the Middle East, with an estimated 200 million women who have been infibulated worldwide [8]. However, despite global and regional propositions towards the eradication of the practice via law and intervention methods, it’s quite saddening to realize that the practice is deeply rooted in some cultures, thereby making its eradication difficult regardless of being tagged internationally as an infringement on human rights [9]. Hence, FGM's ramifications have an eternal effect on the life of any mutilated girl and often health complications such as extended bleeding, cysts, urinary problems, infections, among others. Apart from health-related, moral, and ethical consequences of FGM practice, a report by World Health Organization (WHO) has shown that a massive sum of at least $3.7 million is spent on an annual basis in tackling obstetrics complications [10]. Eighty percent or more of the women undergoing FGM are from Egypt, Ethiopia, Mali, Sudan, Djibouti, and Guinea [11]. This shows that the practice is of high prevalence in Africa.

FGM's persistence is traceable to the fact that those practicing it are not aware of the harm they are causing. Therefore, establishing a Public Health Initiative to eradicate FGM in Africa is considered imperative as newborn females and youth will be safeguarded from undergoing the traumatic ordeal. More so, Africans offshore will be enlightened against the continuation of FGM culture [12].

On the African continent, the traditional components of FGM vary between ethnic regions. Some carry out the procedures on their females between the ages of six and eight while some prefer to cut at birth or before marriage [13]. The mutilation process is undergone alone or in a group of about 40 women or more using the same instruments throughout [14]. The procedure is usually performed in a ceremonial way accompanied by food, music, and gifts. The operators (‘circumcisers’) are often religious leaders with inadequate training or midwives/birth attendants. These circumcisers normally use sharp objects like knives, scissors, clippers, or hot objects [15]. Unfortunately, on most occasions, the environment is not sterile and medical anesthetics are lacking [16]. It is remarkable to note that the resulting wound is sewed with crude instruments such as thorns. In the case of infibulation, the two sides of the labia majora are normally held together with thorns or stitches while the legs may be bound together for up to 40 days [16]. Healing of the wound is aided by the application of an ointment, herbal concoction, or animal excreta, which is thought to help hasten the healing process [17].

The justifications for the persistence of FGM practice differ in many African societies. Some posit that it hinges on cultural identity, which must be safeguarded. Hence, girls pass through such a procedure because it is accepted as an important part of their cultural identity. For others, it is subscribed to out of fear of being stigmatized or rejected by their community. Religious inclination and girls’ marriageability are considered as justification in other regions too [18]. Studies have revealed that in prominent African countries, Christians, Muslims, Animists, and several non-believers in a range of cultures embrace FGM as their religious requirement [19]. However, due to different levels of reasoning among the prominent religious bodies, it was established that a large proportion of mainstream and Protestant Christian groups were found to be against the continuation of the practice whilst Muslims are supportive of its continuation [20].

## Review

Method

Design Protocol and Eligibility Criteria

To achieve a well-coordinated and comprehensive review of this study, pieces of evidence of the best quality were systematically searched for, majorly female genital mutilation with a clear focus on the African continent. Hence, all the studies considered in this review were selected without the restriction of type of study, including the investigation of clinical trials that are relevant to this study as well as systematic reviews and meta-analyses.

However, as the topic implies, studies selected are predominantly stratified in line with female gender but regardless of date, age, tribe, or religious belief. Likewise, no geographical delineation or restriction was established within the confinement of the Africa continent, therefore, all studies relating to Africa in the context of the study are majorly considered.

Information, Source and Search Strategy

For this review, searching for relevant and core materials that are also central to the topic in question was carried out extensively through the databases of PubMed/MEDLINE, Google Scholar, and the references of related articles. In this way, a large number of review articles involving systematic approach, case study report, and meta-analysis were realized in several languages and formats. Of the identified articles, only those written in full-text English language and with no or significantly low level of bias were eventually prioritized. Meanwhile, the search was majorly premised on the studies involving experiences and narratives of women with female genital mutilation to ascertain whether they are victims or even beneficiaries of the act. As such, the search was arranged in keywords like “female genital mutilation”, “female genital mutilation in Africa”, “circumcision”, “experience”, “narrative” and “systematic review”. The aforementioned keywords showed (1) 3,591 peer-reviewed published articles for female genital mutilation, (2) 1,516 peer-reviewed published articles for female genital mutilation in Africa, (3) 38 peer-reviewed published articles for combined keywords for experience and female genital mutilation in Africa, (4) seven peer-reviewed published articles for combined keywords for narrative and female genital mutilation in Africa, (5) six peer-reviewed published articles for combined keywords of narrative, experience and female genital mutilation in Africa, and 10 peer-reviewed published articles for combined keywords of the systematic review and female genital mutilation in Africa.

Results and discussion of findings

Prevalence of FGM in African Countries

Female genital mutilation is a practice that has indeed come of age in the Africa continent. It has a strong sociocultural inclination even though it is oftentimes done secretly and as such underreported, particularly in the last two centuries. The practice is widely embraced by all the prominent religions prevailing in the continent- Islam, Christianity, and traditional worshipers [20]. FGM is performed at different age groups: the week of birth, infancy, before the puberty stage, before the first childbirth and other periods depending on either the location or the particular reason underlying the practice in any society [21]. It is performed individually or sometimes in a group of girls or women. Thus, its extent is commonly estimated although the reported figures have been considered less of what can be observed to be the real extent.

Categorically, the FGM prevalence data are not only for statistic's sake but are essential in serving as a tool for knowing the extent of the practice by policymakers/program managers. Likewise, the number of girls/women who may be at risk of the practice and its trends over time. In the early 1980s, the first estimates of FGM prevalence were carried out on 28 African countries even though it was premised largely on subjective evidence [22]. A decade later, a more sophisticated approach was embraced, which permitted effective systemization of data collection and reporting. This was a population-based survey that gave rise to a collection of data on FGM prevalence at national and sub-national levels concerning population characteristics such as age, religion, ethnicity, residence, etc.; as a result, a module for FGM was included in the prospectus of Demographic and Health Surveys (DHS), as shown in Table 1 below.

**Table 1:** Female Genital Mutilation (FGM) Prevalence in African Countries *MICS (Multiple Indicator Cluster Surveys) *DHS (Demographic and Health Surveys) *EDSF ( Enquête Djiboutienne sur la Santé de la Famille) *PAPFAM (Pan Arab Project for Family Health) *NSPMS (National Social Protection Monitoring Survey) Source: Adapted from UNICEF, 2019. Table 1 is a UNICEF document that does not need permission.

Country	FGM prevalence among girls aged 0 to 14 years (%)	Reference year	Data source
Benin	0.2	2014	MICS
Burkina Faso	13	2010	DHS/MICS
Central African Republic	1	2010	MICS
Chad	10	2014-2015	DHS
Côte d'Ivoire	10	2016	MICS
Djibouti	43	2012	EDSF/PAPFAM
Egypt	14	2015	Health Issues Survey (DHS)
Eritrea	33	2010	Population and Health Survey
Ethiopia	16	2016	DHS
Gambia	56	2010	MICS
Ghana	1	2011	MICS
Guinea	45	2016	MICS
Guinea-Bissau	29	2014-	MICS
		2015	
Iraq	1	2018	MICS
Kenya	3	2014	DHS
Mali	73	2015	MICS
Mauritania	51	2015	MICS
Nigeria	13	2016	MICS
Senegal	14	2017	DHS
Sierra Leone	8	2017	MICS
Sudan	30	2014	MICS
Togo	0.3	2013-2014	DHS
Uganda	1	2011	DHS
United Republic of Tanzania	0.4	2015-2016	DHS
Yemen	15.0	2012	NSPMS

In line with Table 1 above, it was established that the rate of prevalence of FGM was significant in countries like Mali (73%), Gambia (56%), Mauritania (51%), Guinea (45%), and Djibouti (43%). Some other countries like Eritrea, Sudan, and Guinea-Bissau are also characterized by a relatively high rate of prevalence following the above-mentioned ones. This generally implies that the practice of FGM is still extant in African countries.

Prominent Perceptions on the Practice of FGM in African Countries

In most African countries, several perceptions are attached to the adoption of FGM at all levels. Some conceived FGM as a traditional core practice that must be preserved while it is purely a superstitious belief to others, through which their chastity is preserved and they are considered purified [23]. These perceptions of some of the other groups are quite distinct from those as mentioned earlier. Such as a way through which their family honor is preserved, virginity is protected, promiscuity is curtailed, socio-sexual attitude and fertility are enhanced and matrimonial opportunities are increased. Other prominent reasons in some communities are for legal reasons (as an uncircumcised woman cannot inherit property) and prevention of death during childbirth [24]. To this end, for further clarification, these perceptions are hereby emphasized in line with the subdivisions below.

Cultural and Social Perceptions

In Africa, studies and observations have established the fact that the justifications linked to the practice of FGM are quite numerous and in some jurisdictions, compelling. Noteworthy is that the practice varies among several communities, although they are hinged on some common themes such as family honor sake, health, marriageability, and women's status in the communities, among others [25,26,27]. On many occasions, these justifications are presented in a positive manner while less credence is lent to its consequences in order to emphasize its advantages in line with the social-cultural perspective [25]. In fact, in numerous practicing communities and tribes in Africa and the Middle East, FGM is considered a requisite for marriage, and any uncircumcised girl has no chance of being married.

Studies have revealed that FGM in Africa is broadly connected with social status and how girls and their families would be respected in the community [26]. This is evidenced in Sierra Leone culture where it is strictly required to undergo FGM else it will lead to social exclusion and being ousted from the society in general. In this regard, parents are left with no practical choice than to subject their daughters to this act in order to safeguard their images and that of their families. Therefore, the consent of these parents earns them the status of honorable members in the community and their families will be well recognized [26].

Corollary to the aforesaid, a perception elicited in an interview with a woman from Abu-Hasheem village, Egypt, as cited in [27], is as thus: *“Why do you think people here in the village support the practice?” “It is a norm that must be fulfilled so that the girls will be able to protect their honor and that of their family."*

Furthermore, among the FGM practicing groups, the cutting is conceived to be a mark of social and tribal distinction which affords their women and families a social status and value in the society [22]. This corroborates Arusha and Chagga culture in Tanzania, which makes the bride price of circumcised girls higher than their counterparts [28]. FGM/C is also a crude way of preserving girls’ virginity which is a prerequisite for marriage amidst the prominent African cultures. This is traceable to Nigeria, where FGM serves as the sign which is usually verified by mothers-in-law to ascertain the virginity status of the bride before the wedding [29]. Similarly, another justification for FGM is protecting the sexual emotions of girls thereby leading to the preservation of their dignity, morality, and chastity in society. More so, in countries like Somalia and Sudan, bodily cleanliness and beauty are considered as the major reasons why infibulation is carried out on girls.

Religious Perception

In a plethora of studies and scholarly articles, it has been established over time that FGM is of no religious ground, although this fact is partially defeated due to its adoption by few Islamic sects although regarded it as a Sunna-type FGM. However, it has been revealed that no text in the Koran supports the circumcision of the external genitalia of female, thus its prevalence is more apparent among the Sudanese or Nubian Muslims because of its existence in their culture before Islam [30]. In short, the majority of Muslims across the globe do not embrace FGM. Most especially, there is no traceable evidence of it among Saudi Arabians and some parts of African-Islamic nations like Tunisia, Morocco, Libya, and Algeria.

Below are the views of religious leaders with respect to FGM practice as truncated through an interview, cited in [31]:

*“Islamic Shari’a safeguards children and their rights. Hence, those who violate or deprive them of these rights has indeed committed a major sin. As for FGM, it’s a medical issue that we heed and obey as prescribed by doctors. There is basically no text either in Koran or Prophetic Sunna that addresses it’* -Sheikh Mohd Sayed Tantawi, Sheik of Al-Azhar (The Grand Imam).

*“It has been proven to us with genuine religious evidence that there is no rightful Sharia evidence legitimating any form of FGM/C"*. -A statement signed by 30 Sheiks from the eight largest Suffist groups (Muslims) in Sudan (2004).

*"This practice is of no religious base whatsoever. Further, it is morally, practically, and medically groundless. When God created the human being, he made everything good: each organ has its designated function and role. So, why should we disfigure God’s good creation? There is not a single verse in the Bible, nor is there anything in Judaism or Christianity - speaks of female circumcision”.* -Bishop Moussa of Coptic Orthodox Church, and Representative of Pope Shenouda III.

**Figure 1:**
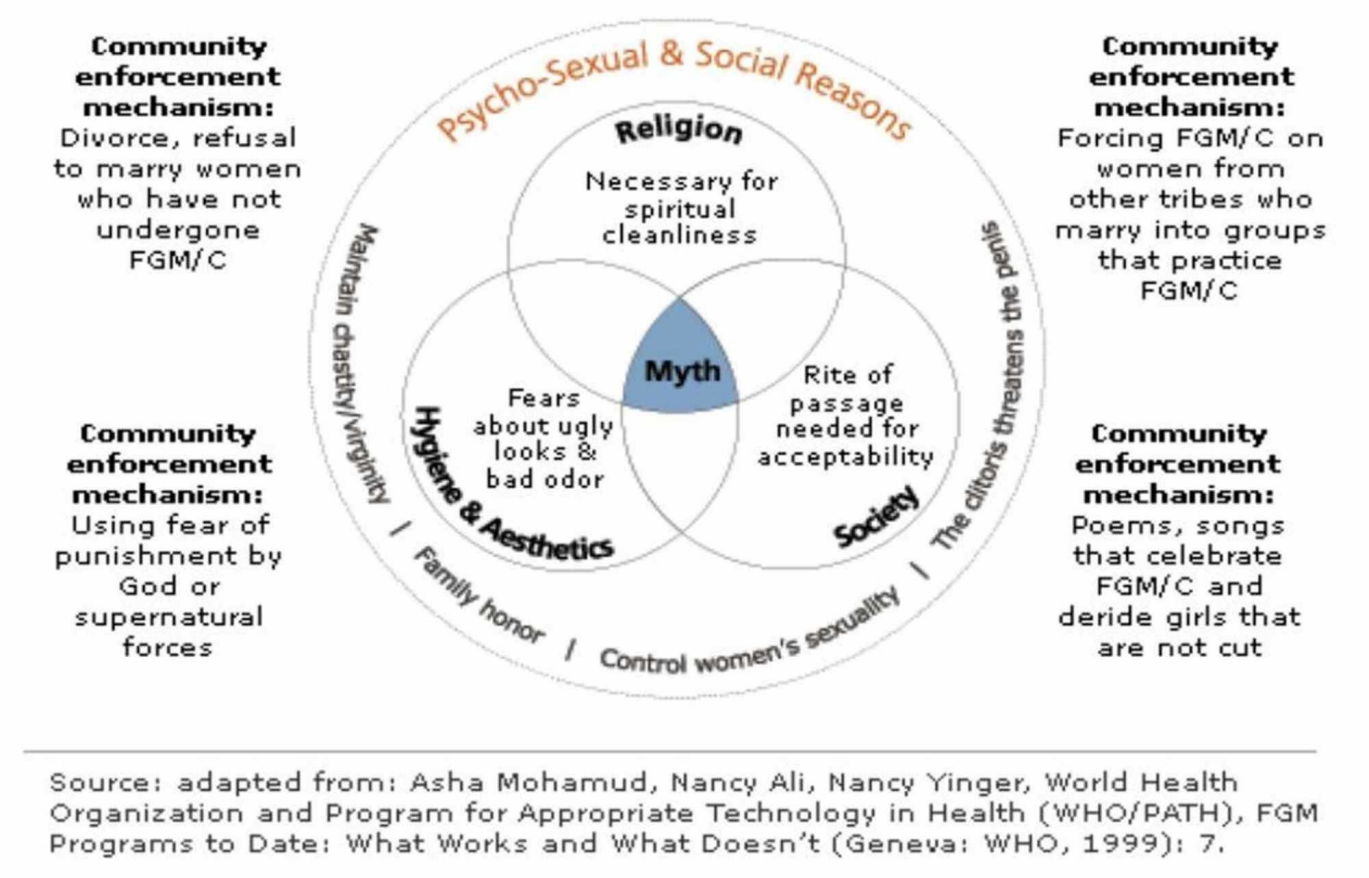
Perceptions of Female Genital Mutilation (FGM) Practice.

Generally, the perceptions of FGM in a more robust yet self-explanatory form is expressed in Figure 1 [32] below:

Consequences of Female Genital Mutilation in Africa

The constant campaign against the widespread practice of FGM is not a matter of subjectivity but a result of adverse effects that have been recorded over the years. FGM has both psychological and physiological complications, which further include short/long term complications [32]. In essence, the consequences are beyond health-inclined ones, as the majority of the victims' mental and psychological stability are often equally disturbed.

Medically, the extent of health complications arising from FGM is largely dependent on the method adopted for the practice [33]. For instance, unsterilized equipment and no medical prescription against adverse effects can potentially cause primary infections like urinary tract infections, staphylococcus, hemorrhaging, and excessive and uncontrollable pains [34]. Specifically in this regard, Type 3 mutilation compared with other types has been revealed to have a propensity of exposing the circumcised women to severe infection such as HIV, clostridium tetani, HSV 2, *Chlamydia trachomatis,* and others [34]. Consequently, the manifestation of these short-term complications will increase the mortality rate due to limited health care available in some African countries.

Although the actual mortality data of girls that have undergone FGM are currently unknown, at least one out of every 500 FGM results in death, even though the general belief in some African societies that FGM protects their girls against sexually transmitted diseases has been debunked by a case-control study carried out in Sudan [34]. The study stressed that, even after the healing of the cutting, the victims often suffer psychological, physiological, and childbirth complications. Therefore, the consequences attached to the act are far beyond what could be overemphasized [34,35].

To this end, prominent among the studies that delved into these consequences are collectively expressed in Table 2 below:

**Table 2:** Consequences of Female Genital Mutilation (FGM) in Africa

Author/Year	Country	Type of study	Focus of the study	Findings	Conclusion
Chibber R et al. (2011) [29]	Egypt	Meta-analysis	Female circumcision, maternal mobility and birth outcome	A survey of 4,800 pregnant women over a 4year period assessed on 95% confidence level specified that there is a positive association between such women and prolonged labor, obstructed labor, cesarean section, and fetal outcome	Female circumcision is linked to adverse maternity issues and psychiatric sequelae. It was therefore recommended to make those women undergo psychiatric and gynecological care
Morison L. et al. (2001) [33]	Gambia	Meta-analysis	long term reproductive health consequences	A cross-sectional community survey of 1,348 women within the age of 15-54 years yielded a result that FGM is highly associated with ethnicity and had a significant rate of prevalence of bacterial vaginosis and herpes simplex virus	An effort to eradicate the practice should be put in place by incorporating the human right approach instead of sole cognizance of damaging health consequences.
Ivazzo C. et al. (2013) [34]	Selected African Countries (unspecified)	A systematic review and clinical evidence	Experiences on FGM and Infections	a study involving 22,052 patients from African countries that have undergone FGM examined posited that the infections faced involved: HIV, chlamydia, trachomatis, Candida Albicans, Neisseria pseudomonas pyocyanin, gonorrhea, treponema palladium, trichomonas vaginalis, HSV-2 and staphylococcus aureus.	A number of infections can ensue after FGM. Therefore a serious step should be taken to eradicate those infections and completely eliminate the practice.
Reyner S. (2004) [35]	Sudan	Literature Review	Health consequences with respect to Gynecological Practice	The comprehensive literature review specified that FGM in women oftentimes leads to bacterial infections, obstetrical complications, and psychological problems.	The study concluded by beseeching the concerned authority on FGM eradication.
El-musharaf S. et al. (2006) [36]	Sudan	Case-control study	Sexually transmitted infections due to FGM	The study used a sampled total number of 222 women of 17-35 years of age attending the antenatal and gynecological clinics. The women were further subdivided into cases with seropositivity for Neisseria gonorrhea, chlamydia trachomatis, or treponema pallidum (26) and control without antibodies (196). hence, out of these cases, those who had undergone a severe type of FGM were 85 compared with 78% of controls. thus, there was no correlation between STIs and extent of FGM	The result indicates that FGM seems to neither cause nor prevent against STIs against the general belief. however, it is considered unreasonable due to the cost implication regardless of maybe it causes any harm or not [37].
Lightfoot-klein et al. (1989) [38]	Sudan	Meta-analysis	Sexual experience and marital adjustment of the female with FGM	The study conducted over a 5year period on 300 Sudanese women on FGM showed that, in spite of female being bound by Sudanese culture to hide their sexual experiences, the number of them that have faced FGM find it difficult to conceal their sexual desire and orgasm as a result.	The study suggested that emotional and mental factors play a crucial role in those clitoridectomized women. Although they were not aware that any option is available to them at all.
Rushwan H. (2000) [39]	Djibouti, Somalia, and northern Sudan	Literature Review	FGM management during pregnancy, childbirth and postpartum	the extensive literature review on FGM practice in those African countries revealed that women with FGM are facing high mortality rate and morbidity from its complications	It was established that the practice should be eliminated through proper awareness and training of personnel
Oyefara J. L. (2014) [40]	Nigeria	Meta-analysis	Psychosocial analysis	Despite the de-medicalization and criminalization of FGM practice in the country, it was still found that the practice was still prevailing as the women sampled clamored for a breakthrough from a psychological problem, sexual abnormalities and unhappiness in marriage as a result of FGM they have undergone	A psychosocial intervention was recommended as a means of improving the wellbeing of affected women. Likewise, the recent increase in practice in the country is advised to be curtailed.
Foldes P. (2016) [41]	Egypt, Somalia, Djibouti, and Yemen	Meta-analysis	Genital Reconstruction Surgery after FGM	the study on 107 randomly selected patients across the study areas whose mean age and cutting are 28.3 and 9.1 respectively revealed that, Before genital reconstruction surgery was carried out on the women with FGM, they were experiencing a high level of sexual abnormalities and excessive pain	The circumcised women are majorly regarded as "victims" and those that require genital reconstruction surgery. Hence, it is psychologically unpleasant.
Rossem R. V. (2015) [42]	Egypt	A meta-analysis on Egypt Demographic and Health Survey (1995-2014)	Women position and attitude towards FGM	The study revealed that women that are literate, educated, and employed usually oppose FGM due to their understanding of the adverse effect of it.	The input of Egyptian women in different social positions has tremendously manifested in the fight against FGM in the country and it should be more enhanced. This could be achieved through Better education and being less traditionalistic.
Oljira. et al. (2016) [43]	Ethiopia	Community cross-sectional study	Experiences between mothers and their daughters	The study which involved 842 mothers and 160 daughters found that 79.5% of mothers and 19% of daughters have undergone FGM. Whereas mothers posited that they considered it beneficial in terms of social acceptance and marriage prospect for their daughters	The study concluded that considering FGM as a beneficial practice to girls/women is largely due to ignorance and lack of knowledge about its implications.
Osterman A. L. et al. (2019) [44]	Senegal	Clinical and epidemiological studies	FGM with respect to invasive cervical cancer	The study involved secondary analysis of combined data from 6 major studies across the subject matter. Hence, it was found that women with FGN are prone to cervical cancer. This was considered reasonable as the data used was time-series data which spans from 1994 to 2012.	The awareness of the health risk surrounding FGM must be carried out extensively and effort must be made to decrease the potential of cervical cancer emanating from FGM practice.
Okeke T.C et al. (2016) [45]	Nigeria	Literature review	Legislations and Health consequences	Review of positions of the World Health Organization (WHO), United Nation Internal Children Emergency Fund (UNICEF), Federation of International Obstetrics and Gynecology suggested that there is no concrete law that bans the act of FGM effectively despite its health implications in terms of obstetrics complication, chronic pelvic infection and sexual difficulties.	FGM is characterized by serious health consequences that have not been put to check by any tangible legislation
Sakeah E. et al. (2018) [46]	Ghana	cross-sectional study	Prevalence and factors associated with FGM among women of reproductive age	Out of 830 women sampled in the study, 61% have undergone FGM with the position that their mother influenced it.	FGM is being practiced majorly by low socioeconomic status women. However, health8 education is highly paramount to eradicate the practice
Puppo V. (2016) [47]	All African countries	Literature Review	Anatomical review and Alternative rites	An extensive literature review on African countries yielded a finding that an average of 3million girls is faced with the risk of FGM each year which exposes them to mental and health challenges consequentially.	Clitoral reconstruction must be carried out on the victims and it must be well established that the practice of FGM only remains a cultural phenomenon than a religious practice.
Gebremicheal K. et al. (2018) [48]	Somali region	A prospective cohort study	sequela of FGM on birth outcomes	The study revealed that circumcised women are characterized by a perineal tear, postpartum blood loss, outlet obstruction, and emergency cesarean section	FGM has a propensity to lead to acute adverse obstetric outcomes. In fact, the extensive the form of FGM, the greater the risk. Therefore, the Adverse obstetric outcomes are quite harmful and as such of long-term effects on health.
Buggio et al. (2019) [49]	African Countries	Narrative review	Psychosexual consequences and impacts on reconstructive surgery	The study found out that girls/women with FGM are more likely to develop psychological disorders such as somatization, post-traumatic stress disorder, anxiety, low self-esteem, and phobia. Likewise, a physiological problem with respect to being sexually stimulated	The psychosexual complications need to be actually curtailed. Equally, a best practice involving a multidisciplinary team must be established to deal with the daunting consequences.
Micheal O. et al. (2016) [50]	Nigeria	cross-sectional study	women perception of sexual disability	the study revealed that women with FGM showed their displeasure about their traumatic experience and plague on their sexual and marital bliss	FGM practice has not been only perpetuating gender inequality but also posing a lot of dangers to its "victims"

In line with health consequences, a study carried out in Djibouti, Somalia, and Sudan on women during pregnancy, childbirth, and postpartum revealed unhealthy complications arising from FGM had increased both mortality and morbidity among those women [32]. Likewise, in Gambia, Sudan, Egypt, Somali, Nigeria, and other parts of Africa, studies have revealed that high prevalence of FGM has posed several health challenges such as bacteria vaginosis, herpes simplex virus, Neisseria gonorrhea, Chlamydia trachomatis or treponema pallidum, trichomonas, Candida albicans, Pseudomonas pyocyanin, vaginalis Staphylococcus aureus, HSV-2, prolonged labor, obstructed labor, cesarean section and fetal outcome, obstetrics complication, perineal tear, postpartum blood loss, chronic pelvic infection, and sexual difficulties [32,33,34,35,37,45,50]. Generally, these health consequences are gynecological; linkable to infection, urinary symptoms and infertility, scarring/keloid, menstrual difficulties, and obstetric complications during pregnancy, labor as well as postpartum period. 

Concerning long-term complications, perhaps the most severe one is the forming of keloid scar tissue over the cut area which often leads to anxiety and shame for the victim [38]. Resultantly, neuromas may equally submerge as a result of the entrapped nerves around the scar, thereby leading to pain, especially in the process of intercourse [38]. For instance, there has been a long record of mortality rate for infibulated women due to the complications that usually arise during childbirth [32].

Similarly, the reviewed studies equally established that FGM entails psychological, psychosocial, and psychosexual effects, although there is a few quality evidence to adequately back it [49]. Some studies identified effects such as anxiety, post-traumatic disorder, and depression, perhaps as a result of shame arising from social victimization [38]. In the study of Behrendt and Moritz, where the mental health status of 47 Senegalese women was sampled via clinical interview, it was established that 23 among them had undergone FGM. Thus, a high prevalence of psychiatric problems such as memory loss (47.9%) and post-traumatic disorder (30.4%) was found in women with FGM. Therefore, the prevalence of problems associated with mental health in circumcised women was statistically significant. More so, FGM has also been traced to sexual dysfunction as the clitoris may be removed in the process. However, studies on sexual function post-FGM are relatively few and frequently devoid of standardized and appropriate questionnaires and control groups, which could serve as reliable evidence for valid inference.

In a study comprising 300 Sudanese women on the sexual experience and marital adjustment of the female with FGM, it was revealed that mental and emotional effects glaringly manifest in the lives of those clitoridectomized women [38]. This study commensurates the findings from a narrative review of psychosexual consequences and a meta-analysis study on Nigerian women based on their psychosocial analysis. The former posited that women with FGM are more prone to developing psychological disorders such as phobia, anxiety, somatization, self-esteem, and post-traumatic stress disorder and equally physiological problem in respect to being sexually stimulated, while the latter revealed that FGM creates psychological problems, sexual abnormalities, and unhappiness in marriage [48,49].

Limitations

Like other studies, this study has limitations. First, taking full consideration and premising the review on the whole of African nations, even regardless of credence being duly lent to the prominent parts, is a concern although measures were taken to achieve all-inclusiveness. Still, some parts of the nation or ethnic groups might not have been adequately taken into account. More so, the review focused mostly on the adverse effects of FGM on health, psychosocial, and psychological areas with little indication to the solution in a real sense. Also, the analyses do not evaluate the effect of legal bans on FGM on societal attitudes towards FGM. However, one can hope that the statutory prohibition on FGM will catalyze its de-legitimation as with other related harmful practices. However, the review captured narratives and experiences of mutilated women from several germane studies with in-depth analysis and extensive coverage.

## Conclusions

Towards harnessing the feelings and thoughts of African women about the practice of FGM in their respective territory, the findings, which hinged on the cultural, social, and partly religious ideology about the practice, are indicative of the likely rationale behind the continued practice of FGM in all African regions. Little wonder the practice persists despite the ongoing global campaigns against it. Unfortunately, mothers, who are responsible for protecting their children, are in good faith subjecting their daughters to this detrimental practice for fear of being victimized or ousted from the community. Therefore, it is important to ensure that culture, myths, and misconceptions associated with the practice are dismissed, and proper enlightenment is sufficiently channeled because women are consistently victims of this barbaric practice. This victimization can be viewed from the angles of gynecological, psychological, psychosocial, and psychosexual implications which were realized to be dangerous to the existence of the circumcised women. Thus, FGM is an outdated practice that perpetuates gender inequality in society and endangers women by exposing them to disabling consequences and complications. In line with this, as revealed by past studies, proper awareness about FGM's consequences and, most notably, female empowerment through girl-child education beyond peripheral will have potential in reducing female circumcision.

Currently, there is a need for a collective effort at all levels to deal with this disabling cultural practice. This could be achieved by criminalizing the practice, providing both adequate medical and psychosocial aids for its victims, and encompassing traditional and religious heads in campaigns against the practice and anti-FGM initiatives. More so, to curtail the prevalence of FGM in a real sense, the approaches have to be holistic, community-based, and feature human rights education at all levels. Resultantly, there would be a drastic social transformation among individuals and families in African communities, which will potentially end FGM and quickly motivate the remainder of the intermarrying population. With these, there would be a drastic reduction in the prevalence of FGM in Africa and the narratives will change in years to come.
